# Assessing COVID19-related anxiety in an Egyptian sample and correlating it to knowledge and stigma about the virus

**DOI:** 10.1186/s43045-021-00094-9

**Published:** 2021-03-11

**Authors:** Samah Hamed Rabei, Wafaa Osman Abd El Fatah

**Affiliations:** 1 Psychiatry, Faculty of Medicine, Helwan University, Cairo, Egypt; 2 Psychiatric Nursing, Faculty of Nursing, Helwan University, Cairo, Egypt

**Keywords:** COVID19, Anxiety, Knowledge, Stigma

## Abstract

**Background:**

COVID19 public health crisis has led to extensive anxiety regarding spread of virus. Aim of study is to assess COVID19-related anxiety in Egypt and correlate it to knowledge and stigma.

**Results:**

Online questionnaire, 17–20 April 2020, had 218 Egyptian respondents to a socio-demographic questionnaire and Generalized Anxiety Disorder Scale (GAD-7). The present study revealed that 21.2% of the respondents were experiencing severe anxiety, 34.8% moderately severe anxiety, 25.2% moderate anxiety, and 18.8% mild anxiety. Women have more rates of severe anxiety. People who get online knowledge about COVID19 have least rates of severe anxiety. 51.8% think having the virus is stigmatizing. Knowledge and stigma are insignificantly inversely correlated to anxiety scores

**Conclusion:**

Online scientific health education is necessary to reduce anxiety.

## Background

Emergency Psychiatry encompasses disaster psychiatry, a part of which is epidemic and pandemic psychiatry. This should address three targets: (1) public suffering uncertainty and isolation consequences, (2) medical workers and families who face stigma besides, and (3) patients who face biological consequence besides and besides.

### Literature about mental health during pandemics remains scanty

The earliest studies reported 22% lifetime prevalence of *depression* in human immunodeficiency virus (HIV) patients in the early 1980s. Mental health of medical workers, patients in isolation, and survivors were studied for severe acute respiratory syndrome (SARS) caused by coronavirus in China in 2003. Post-traumatic stress disorder (PTSD) was recorded in 20% of medical workers. WHO Mental health policy was accused of creating “panic-demic” peddling unproven vaccines during the Swine flu H1N1 in Mexico in 2009. *Zika* in Uganda in 2014 appeared in social media, perhaps for the first time in history, and this allowed social researchers to study the public sentiment, also known as the *emotional epidemiology*. It indicated that 4 out of 5 posts on Zika on social media were accurate; yet, those that were “trending” were “fake news” [1].

Novel coronavirus disease in 2019 (COVID19) is a zoonotic beta-coronavirus that was first reported and then became widespread within Wuhan, the capital city of Hubei Province of China. Then, the disease rapidly spread throughout China and elsewhere, becoming a global health emergency [2]. Clinical presentation of COVID19 greatly resembled viral pneumonia such as SARS and MERS. Most cases are mild cases (81%) whose symptoms were usually self-limiting and recovery occurred in 2 weeks. Severe cases progressed rapidly with acute respiratory distress syndrome (ARDS) and septic shock and eventually ended in multiple organ failure [3]. World Health organization stated in its COVID19 situation report -198, that global cases exceeded 18 million cases and deaths exceeded half a million [4].

To face this existential anxiety, problem solving parallels emotional coping. Problem solving includes trying to find medications and vaccines, prepare serums, and minimize infection through preventive policies like (a) travel restrictions: protective sequestration, quarantine, and sanitary cordon; (b) shelter-in-place and social distancing: cancel mass gatherings, school, and workplace closures; and (c) case separation and staff high isolation (gloves, eyewear, waterproof gown, N95 respirator). These policies lead to *isolation and lack of emotional support* (separation from loved ones, masked facial expression, no skin contact) beside the baseline *uncertainty* about getting sick, recovering, surviving, or loosing loved ones [5–7].

Emotional coping can be externally controlled to a great extent. Mood and behavior spread in populations by exposures to surrounding people or through media. We imitate most outspoken, encouraging, in similar situation persons [8]. This creates an *emotional and behavioral contagion (public panic or mass hysteria)* shadowing the original pandemic. In 2256 adults poll commissioned by National Health Council, medical news were influential, as half viewers took some health actions [9].

When problem solving and emotional coping fail, there are expected high rates of PTSD, anxiety, depression, or substance abuse among the public during pandemics [6].

Disseminating *solid evident epidemiological facts* in outbreak is the best countering to anxiety-inducing uncertainties, rumors, and speculations. We need to address emotional pandemic accompanying actual outbreak [10, 11]. Ignorance and fear can lead to massive uncontrollable behaviors that have deleterious effects for entire societies. If pandemic is triggered, in bioterrorism or biological war, emotional epidemiology becomes a target in the era of social media with organized hostile malicious feeds as rumors, half-truths, and “fake news” to maximize impact by heavily manipulating pre-existing and developing public attitudes [12].

Managing emotional epidemics shadowing microbial epidemics is essential especially in biological warfare and bioterrorism as a mean of defense in psychological wars.

### Significance of the study

Psychiatric studies in pandemics remain scanty. This study assesses COVID19-related anxiety in Egypt and correlates it to knowledge and stigma in Egypt.

### Hypothesis

Knowledge about COVID19 decreases anxiety and stigma related to it.

## Methods

### Design

Descriptive analytical research

### Setting, timetable, and sampling

Online self-reported questionnaire. The link was sent in April 2020 to all WhatsApp groups accessible to researchers and on their Facebook pages. Members of these groups and followers were encouraged to send the link to their friends and family members who are interested in giving feedback. *Sample size* is calculated using Epi-Info program version 6 assuming 95% confidence interval, 80% power of test; accordingly, the following equation is used: *n* = (*z*/*e*) *2 (*p*) (1 − *p*)

where *n* = the sample size, *p* = the expected prevalence, *z* = the critical value 1.96, and *e* = the margin of sample error tolerated 0.05

The expected prevalence according to Hyland et al. is 20% [13]. Therefore, the sample size was calculated to be 218 participants.

### Tools

1. *Socio-demographic data*: age, sex, address, level of education, and source of knowledge about the illness (academic courses, newspapers, television, non-academic online sites, and personal acquaintances)
2. *Semi-structured knowledge and stigma assessment* according to respondents’ satisfaction with their own level of knowledge and their perspective of the illness as a stigma or not
3. *Generalized Anxiety Disorder Scale (GAD-7)*: 7-item Generalized Anxiety Disorder Scale inquires the frequency with which respondents suffered from these symptoms within the last two weeks using a 4-item Likert rating scale ranging from 0 (not at all) to 3 (almost every day), such that the total score ranges from 0 to 21. 0–5 = mild, 6–10 = moderate, 11–15 = moderately severe anxiety, and 15–21 = severe anxiety [14].

### Ethical considerations

Agreements for participation were taken after explaining the purpose of the study, and before data collection. They were given an opportunity to refuse to participate and they were notified that they can withdraw at any stage of research without giving any reason. Also they were assured that the information given will remain confidential and used for the research purpose only.

### Statistical analysis

The collected data were organized, tabulated, and statistically analyzed using the statistical Package for the Social Sciences, SPSS version 16. For quantitative data, the range, mean, and standard deviation were calculated. Pearson correlation was used to correlate variables.

## Results

### Socio-demographics

More than half of the studied respondents are in the age range 18–35 (*n* = 121, 55.5%), females (*n* = 122, 56%), and have university education (*n* = 114, 52.3%). All of the samples are studying in different levels of education, and only 68 of them (31.2%) work part-time jobs while studying. More than 95% of them live in urban areas in 13 upper and lower Egyptian governorates (Cairo, Giza, Kalyoubia, Dakahlia, Alexandria, Behaira, Kafr Al Sheikh, Monofia, Gharbia, Fayoum, Menya, Assuit, Aswan) (see Table 1).

**Table 1:** Socio-demographics, knowledge, and stigma among studied subjects (*n*= 218)

Type	Subclass	No.	%	Type	Subclass	No.	%	Type	Subclass	No.	%
1. Age	18–25	121	**55**	**5.** Know about **symptoms**	Good	119	**54.6**	**8. Know** corona **case**	Yes	13	**6**
	26–35	61	**28**		Moderate	97	**44.5**		No	205	**94**
	>35	36	**17**		Poor	2	**0.9**	9. **Size of problem**	Big	72	**33**
2. Sex	Male	96	**44**	**6.** Know about **improvement**	Good	39	**17.9**		Moderate	101	**46.3**
	Female	122	**56**		Moderate	146	**67**		Small	45	**20.7**
3. Education	School	58	**26.6**		Poor	33	**15.1**	**10.** Expect **control** over corona	Soon	52	**23.8**
	University	114	**52.3**	**7. Source of knowledge**	Medical specialists	14	**6.4**		Not know	108	**48**
	Masters	13	**6**		Newspapers	33	**15.1**		Not soon	68	**18.2**
	Doctorate	33	**15.1**		TV	35	**16.1**	11. See corona a **stigma**	Yes	113	**51.8**
4. Residence	Rural	9	**4.6**		Online news	119	**54.6**		No	**105**	**48.2**
	Urban	208	**95.4**		Personal 0	17	**7.8**				

### Knowledge and stigma

**Fig. 1:**
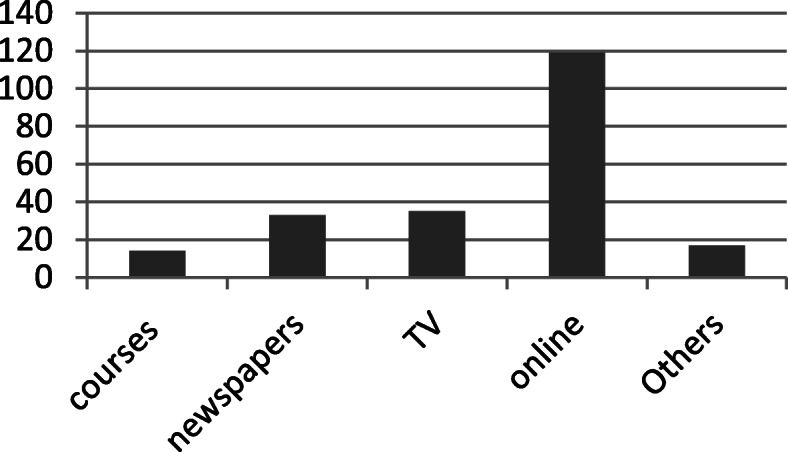
Sources of knowledge about corona

More than half of the respondents rate their knowledge about COVID19 symptoms as good (*n*=119, 54.6%) and almost two thirds rate their knowledge about improvement symptoms as moderate (*n*=146, 67%). More than half of the respondents get their knowledge about COVID19 from online news (*n*=54.6%) (see Fig. 1). Almost 94% of them have not seen case of corona personally. Almost half of the respondents see COVID19 as a moderate problem (*n*=101, 46%) and do not know when it would be controlled (*n*=105, 48%). More than half of the respondents find COVID19 stigmatizing (see Table 1).

### Anxiety levels

**Fig. 2:**
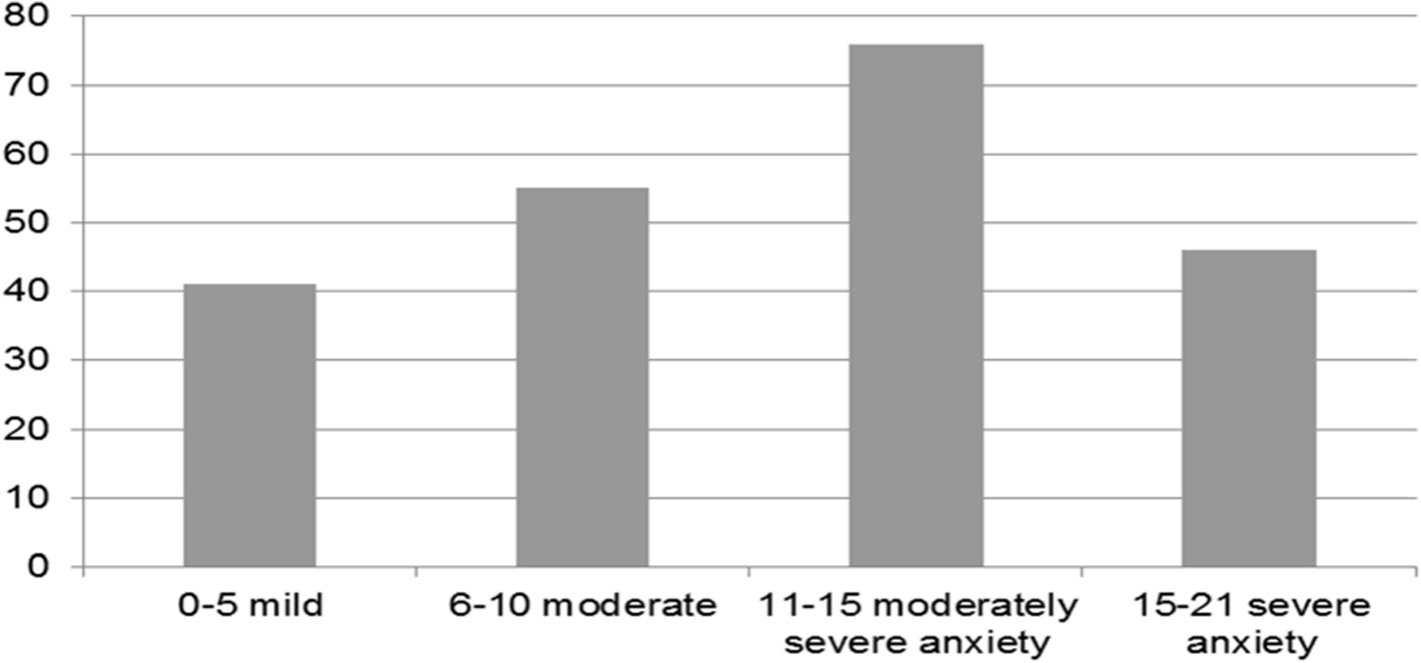
Anxiety levels as to GAD7

One fifth of the respondents (*n*=46, 21.2%) experience severe anxiety, more than one third experience (*n*= 76, 34.8%) moderately severe anxiety, more than a quarter experience (*n*=55, 25.2%) moderate anxiety, and less than one fifth experience (*n*=41, 18.8%) mild anxiety (see Table 2 and Fig. 2).

**Table 2:** Total score of General Anxiety Disorder (GAD-7) among studied subjects (*n*= 218)

Anxiety level	No	%
0–5 mild	41	18.8
6–10 moderate	55	25.2
11–15 moderately severe anxiety	76	34.8
15–21 severe anxiety	46	21.2
Total	218	100.0

### Correlations of socio-demographics, knowledge, and stigma to score of anxiety on GAD7

Rural residence, stigma, satisfactory knowledge about the symptoms of COVID19, and improvement of these symptoms are the factors that were inversely correlated to anxiety scores measured by GAD7; yet these inverse correlations are not statistically significant (see Table 3). Rural residents, people who are satisfied with their knowledge about COVID19, and people who find covid19 stigmatizing have lower scores of anxiety.

**Table 3:** Correlations of socio-demographics, knowledge, and stigma to score of anxiety on (GAD7)

Socio-demographics, knowledge, and stigma	***r*** (Pearson’s correlation)	***P*** value
1. Age	0.045	0.510
2. Sex	0.266	**0.000**
3. Education	0.059	0.384
4. Residence	−0.013	0.854
**5.** Know about **symptoms**	−0.005	0.942
**6.** Know about **improvement**	−0.061	0.374
**7. Source of know-ledge**	0.206	**0.002**
**8. Know** corona **case**	0.000	0.999
9. **Size of problem**	0.063	0.351
**10.** Expect **control** over corona	0.107	0.116
**11.** See corona a **stigma**	−0.013	0.847

There is a high statistical significance of the correlation between sex and scores of anxiety (see Table 3). Women have higher rates of severe anxiety (29/122) than men (18/96).

There is a high statistical significance of the correlation between source of knowledge about covid19 and scores of anxiety (see Table 3). Almost half of those who get their knowledge from newspaper (17/33) have severe anxiety. Almost one third of those who get their knowledge from personal communication (6/17) have severe anxiety. Almost one fifth of those who get their knowledge from medical specialists and television had severe anxiety (3/14, 7/35 respectively). Less than one sixth of those who get their knowledge from online news have severe anxiety (17/119). Severe anxiety is least among those who get their knowledge online.

### Correlations of knowledge to stigma

Stigma is correlated to knowledge about the symptoms of covid19 and inversely correlated to knowledge about improvement symptoms of covid19, yet these correlations are not statistically significant. Rather interesting is that focusing on symptoms of improvement rather than symptoms of illness can inverse the correlation to stigma. It shows how the way of displaying data in a positive way can change the attitude of people to harbor lower levels of stigma when speaking about symptoms in the context of improvement and not the context of deterioration (see Table 4).

**Table 4:** Correlations of knowledge to stigma

Socio-demographics, knowledge, and stigma	***r*** (Pearson’s correlation)	***P*** value
Know about **symptoms**	0.6	0.381
Know about **improvement**	−0.05	0.465

## Discussion

The hypothesis of this study has been fulfilled as follows:

One fifth of the respondents (*n*=46, 21.2%) experience severe anxiety, more than one third experience (*n*= 76, 34.8%) moderately severe anxiety, more than a quarter experience (*n*=55, 25.2%) moderate anxiety, and less than one fifth experience (*n*=41, 18.8%) mild anxiety (see Table 2 and Fig. 2). Hyland et al. [13] report rates of anxiety in *Ireland* during COVID19 pandemic to be 20%. Interestingly, Hyland et al. assessed anxiety using GAD-7 which is used in this study [13]. Huang et al. [15] report anxiety rates in *China* during COVID19 pandemic to be 35.1%. Huang et al. used the National Internet Survey on Emotional and Mental Health (NISEMH) to assess anxiety, depression, and sleep quality [15]. Stanton et al. [16] report anxiety in *Australia* during COVID19 to be 3.4%. They used Depression, Anxiety and Stress Scale (DASS 21) [16]. Al Sharji [17] reports anxiety during COVID19 in Kuwait to be 53.7% using GAD7 used in this study. Al Omari et al. [18] studied a sample from Jordan, Egypt, Oman, Emirates, Saudi Arabia, and Iraq using DASS 21 and reported anxiety rates to be extremely severe in 11.2%, severe in 6%, moderate in 16.2%, and mild in 7% in the whole sample. In the Egyptian respondents, anxiety rates were extremely severe in 13.7%, severe in 8.8%, moderate in 22%, and mild in 7.1% [18]. The difference in results regarding the Egyptian respondents in Al Omari et al.’s [18] study and this study could be explained by the difference in sensitivity and specificity between tools used in each study: GAD7 and DASS21.

More than half of respondents (55%) are in the age group 18–25. High levels of anxiety among respondents could be explained by Chen et al. (2005) [19] and Yang et al. [20] who discussed that the continuous spread of the outbreak led to strict isolation measures and delays in starting schools, colleges, and universities across which influenced the mental health of children, and older adults.

There is a high statistical significance of the correlation between sex and scores of anxiety (see Table 3). Women have higher rates of severe anxiety (29/122) than men (18/96). This agrees with Al Omari et al. [18] and Al Sharji [17]. This could be explained by higher anxiety rate in females discussed by Mclean et al. [21].

There is a high statistical significance of correlation between source of knowledge about COVID19 and scores of anxiety (see Table 3). Colet et al. (2018) highlighted that the risk of acquiring and spreading infection must be minimized through knowledge about infection control to diminish infection risk. Contemporary research has investigated and recognized the critical role played by infection prevention through proper provision of knowledge [22].

### Correlations of knowledge to stigma

Stigma is correlated to knowledge about the symptoms of COVID19 and inversely correlated to knowledge about improvement of COVID19. But these correlations are not statistically significant (see Table 4). This is evident in how *WHO* provides expert guidance and answers to public questions, to help people manage fear, stigma, and discrimination during COVID19 as new diseases have the most devastating effects globally; its emergence and spread cause fear which is the breeding ground for hatred and stigma. It is vital to avoid this stigma as it can make people hide their illness and not seek health care immediately [23]. Minimizing stigma could be done by introducing data in a professional way that understands the emotional state and needs of audience. In this study, a rather interesting thing is that focusing on symptoms of improvement of illness had inverse or negative correlation to stigma unlike symptoms of developing the illness which had a positive correlation to stigma. It shows how the way of displaying data in an optimistic way without messing with facts can change the attitude of people to harbor lower levels of stigma when speaking about symptoms in the context of improvement and not the context of deterioration (see Table 4). When addressing the public data tailored and displayed honestly but still in a context that enhances both problem solving and emotional coping of the public.

## Conclusion

*In the light of the current study results, it could be concluded that:*

1. Emotional pandemics shadow microbial pandemics
2. Emotional contagion could be used in psychological war to aggravate biological war attacks
3. Psychiatric services on 1ry, 2ry, and 3ry basis are no luxury in combating pandemics
4. Psychiatric research and management programs in pandemics is scanty, lacking although essential
5. Disseminating solid evident epidemiological *facts* in outbreak is the best countering to *anxiety*
6. Countering anxiety is vital to avoid this *stigma*
7. Stigma makes people hide their illness which renders disease *control* impossible

### Recommendations

*From the previous findings, the following recommendations are suggested:*

1. Research plans: further research to assess, analyze, and test effectiveness of future designed mental health polices to create a *loop of national auditing* of mental health services
2. *Clinical application*: development of psychiatric services that combat mental illnesses on 1ry, 2ry, and 3ry levels
3. *Educational Policies*: development of educational programs for all health care providers in hospitals that target *mental and social* wellbeing of *themselves, patients, their families, and the public during, after, and in preparation* to other potential pandemics
4. *Approach to media and public*: provision of simplified evident data tailored to enhance both problem solving and emotional coping of the public to create a science-oriented and psychiatry-oriented culture among people in general
5. *Approach to policy*: cooperation to create sufficient psychiatric r*esearch, services, education, and awareness* that meet the need of community and raise the quality of life of citizens

## Data Availability

Applicable
